# Protocol for non-invasive genotyping of axolotls using skin swabbing

**DOI:** 10.1016/j.xpro.2026.104760

**Published:** 2026-08-04

**Authors:** Laura Isabella Arbanas, Aida Rodrigo Albors

**Affiliations:** 1 Centre for Regenerative Medicine, Institute for Regeneration and Repair, The University of Edinburgh, Edinburgh EH16 4UU, UK; 2 Institute for Stem Cell Research, School of Biological Sciences, The University of Edinburgh, Edinburgh EH16 4UU, UK

**Keywords:** Developmental biology, Model Organisms, Molecular Biology

## Abstract

Fin clipping is the standard method for sampling axolotl DNA for genotyping, but it requires anesthesia and removing a piece of tissue. Here, we present a non-invasive protocol for axolotl DNA sampling and genotyping using skin swabbing. We describe the steps for extracting DNA from skin cells collected with cotton swabs and then detail how to sex genotype axolotls using PCR and gel electrophoresis. This protocol removes the need to cause an injury before regeneration experiments and improves axolotl welfare.

## Highlights

• Guidance on skin swabbing axolotls for DNA sampling
• Steps for robustly extracting DNA and sex-genotyping axolotls by PCR
• Instructions for reading axolotl sex genotypes from gel electrophoresis

## Abstract (editor highlights)

Publisher’s note: Undertaking any experimental protocol requires adherence to local institutional guidelines for laboratory safety and ethics.

## Graphical abstract

**Figure:**
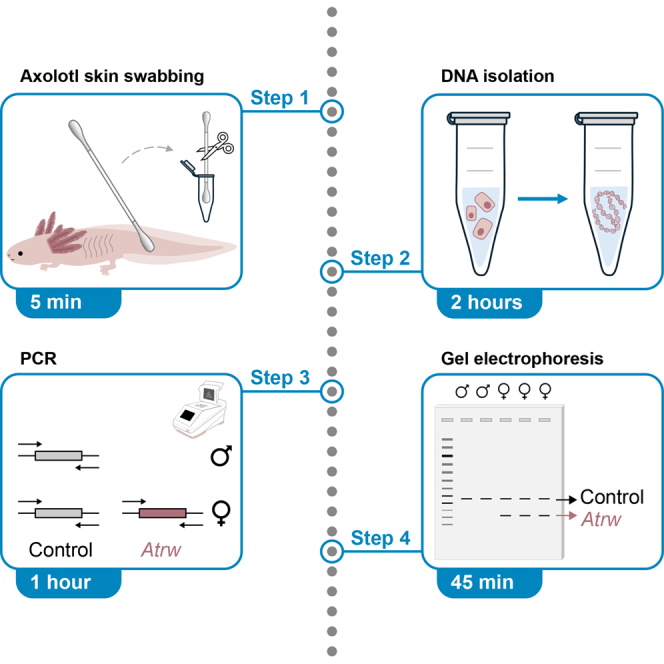

## Before you begin

Axolotls (*Ambystoma mexicanum*) have the remarkable capacity to regenerate complex body parts including limbs,^1^^,^^2^^,^^3^ tail,^4^^,^^5^^,^^6^ spinal cord^7^^,^^8^^,^^9^ and large parts of their brain.^10^^,^^11^^,^^12^ How axolotls achieve full regeneration remains a fundamental question in biology and one with the potential to inform new strategies for tissue regeneration in humans. The sequencing of the axolotl genome^13^^,^^14^^,^^15^ and recent advances in genetic and molecular tools^2^^,^^16^^,^^17^^,^^18^^,^^19^^,^^20^^,^^21^ have made this question increasingly tractable, and axolotl research facilities are growing in number worldwide.^22^ In this context, determining the sex of axolotls early and accurately is essential for colony management, breeding and investigating sex-specific differences. Sex in axolotls is set at fertilization by a ZW/ZZ chromosomal system, in which females are heterogametic (ZW) and males are homogametic (ZZ).^23^^,^^24^^,^^25^ However, sex cannot be identified visually until sexual maturity (12-15 months of age), and therefore earlier sex determination in axolotls relies on genotyping. The current standard for sampling axolotl DNA for sex genotyping is surgical fin clipping under anesthesia. Although axolotls regenerate, this is an invasive procedure that causes an injury and triggers a cellular response that can confound regeneration experiments. Skin swabbing is a non-invasive alternative that has been successfully used for DNA sampling and genotyping in other aquatic species, including zebrafish and sticklebacks.^26^^,^^27^ In this protocol, we describe how to use cotton swabs to collect DNA from axolotls without the need for anesthesia or the removal of a piece of tissue. We also detail how to extract and purify DNA from axolotl mucus and skin cells for PCR-based genotyping using two well-validated primer pairs^25^^,^^28^: one targeting a female-specific gene (*Atrw*) and one targeting a gene present in both male and female axolotls (*Rcc2*), as an internal positive control. We then provide instructions on how to use gel electrophoresis to visualize the PCR products and assign sex unambiguously. Our protocol works reliably across larval (2.5 cm snout-to-tail tip), juvenile (4 cm) and adult (25 cm) axolotls. We demonstrate our approach for sex genotyping, but we expect it to be directly applicable for genotyping transgenic axolotls.

All axolotl experimental procedures were approved by the Animal Welfare and Ethical Review Body (AWERB) of The University of Edinburgh and the Home Office (project license PP9199678) and complied with local and national regulations. Skin swabbing is not a regulated procedure, but surgical fin clipping is and requires specific project and personal licenses in place.

### Innovation

Traditional DNA sampling for genotyping in axolotls relies on fin clipping, an invasive procedure that requires anesthesia and tissue removal. In this protocol, we present an alternative method for axolotl DNA sampling and genotyping that offers experimental and logistical advantages while directly supporting the 3Rs principles of refinement and reduction. In light of a recent study showing that amputation triggers a body-wide and long-lasting cell proliferation response in axolotls,^29^ skin swabbing represents a key advantage over fin clipping because it does not trigger an injury-like response that can confound regeneration studies (see expected outcomes). This makes it possible to genotype and sex axolotls prior to regeneration experiments to, for example, investigate sex-specific differences. Although low and inconsistent DNA recovery has been reported as a limitation of skin swabbing in other amphibian species,^30^^,^^31^^,^^32^^,^^33^ the naturally wet skin of axolotls and our refined DNA extraction yield sufficient DNA across life stages, from 2.5 cm larvae to 25 cm adults. As a non-regulated procedure, skin swabbing improves axolotl welfare and can be implemented in research facilities awaiting the project and personal licences required for regulated procedures. This makes our protocol a practical and welfare-conscious alternative to fin clipping that is immediately accessible to the global axolotl research community.

### Institutional permissions

All axolotl experimental procedures were performed according to The University of Edinburgh and Home Office regulations under project license PP9199678.

## Key resources table

**Table:** 

REAGENT or RESOURCE	SOURCE	IDENTIFIER
**Chemicals, peptides, and recombinant proteins**		

UltraPure^TM^ Tris Buffer (powder format)	Thermo Fisher Scientific	Cat# 15504020
Hydrochloric acid 37%	Sigma-Aldrich	Cat# 320331
Sodium chloride	Sigma-Aldrich	Cat# S3014
Sodium dodecyl sulfate	Sigma-Aldrich	Cat# 436143
Acetic acid glacial, 99+%, extra pure	Thermo Fisher Scientific	Cat# A/0360/PB17
UltraPure^TM^ 0.5M EDTA, pH 8.0	Invitrogen	Cat# 15575020
Isopropanol, 99.5% for molecular biology, DNA	Thermo Fisher Scientific	Cat# 327270010
Ethanol Absolute, 99%	Sigma-Aldrich	Cat# 1070172511
Nuclease-free Water (not DEPC-Treated)	Invitrogen	Cat# AM9937
Terra PCR Direct Polymerase Mix	Takara	Cat# 639271
UltraPure Agarose	Life Technologies	Cat# 16500500
SYBR Safe DNA Gel Stain	Invitrogen	Cat# S33102
100 bp DNA ladder and 10X Blue Juice	Invitrogen	Cat# 11538766
QuickLoad® 100 bp DNA ladder and 6X gel loading dye	Biolabs	Cat# N0551S
EdU (5-ethynyl-2′-deoxyuridine)	Invitrogen	Cat# A10044
Click-iT™ EdU Cell Proliferation Kit for Imaging, Alexa Fluor™ 488 dye	Invitrogen	Cat# C10337
Donkey Serum	Bio-Rad	Cat# C06SB
PBS pH 7.4 (10X)	Gibco	Cat# 70011044
Triton-X100	Merck	Cat# 9036-19-5
Formaldehyde, extra pure, solution 37–41%	Fisher Chemical	Cat# 10630813
SlowFade Gold antifade reagent	Invitrogen	Cat# 11590676
SuperFrost Plus Adhesion slides, 25x75x1mm	Epredia	Cat# J1800AMNZ
OCT embedding matrix	CellPath	Cat# KMA010000A

**Experimental models: Organisms/strains**		

*Ambystoma mexicanum*: white (d/d); male and female; 2.5-20 cm snout-to-tail tip (1-12 months of age)	IRR axolotl research facility (Edinburgh, UK)	N/A

**Oligonucleotides**		

Axolotl-control-genotyping primers: fwd 5′- GGTTTTAATTGTGATCAGTGGTACAG-3′ rev 5′-AAAGAAATAAATAGGGCAAACAACAC-3′	Keinath *et al.*, 2018^25^	N/A
Axolotl-female-genotyping primers: fwd: 5′-AACCCAATACAAAAGGTAAACATGTAG-3′ rev: 5′- AGAAAGAGAAATTGGGCTTACTTTAAC-3′	Keinath *et al.*, 2018^25^	N/A

**Software and algorithms**		

Fiji	Schindelin *et al.*, 2012^34^	N/A
GraphPad Prism 11	www.graphpad.com	N/A
Adobe Illustrator	Adobe	N/A

**Other**		

Sterile Cotton Series, CleanTips Swabs	Texwipe	Cat# 15823886
PCR Tube Strips 0.2 ml	Eppendorf	Cat# 951010022
DNA LoBind Tube 1.5 ml	Eppendorf	Cat# 0030108051
PTC Tempo Thermal Cycler	Bio-Rad	Cat# 12015309
Mupid One Electrophoresis System	Eurogentec	Cat# MU-0041+
ThermoMixer	Eppendorf	Cat# 5382000031
Minicentrifuge	Starlab	Cat# S8030-5000
Refrigerated Centrifuge 5425R G, GB-plug, Rotary Knobs, w/Rotor FA-24x2	Eppendorf	Cat# 5405000263
Vortex Genie 2	Scientific Instruments	Cat# SI-0266
NanoDrop ND-1000 UV/VIS Spectrophotometer	Thermo Fisher Scientific	N/A
ChemiDoc™ MP Imaging System	BIO-RAD	Cat# 734BR-3742
Swann-Morton Disposable Sterile Scalpel No. 10	Swann-Morton	Cat# 1129777
Hamilton 100 μL microliter syringe, Removable Needle	Hamilton	Cat# 7638-01
27 gauge, Small Hub Removable Needle	Hamilton	Cat# 7803-01
Epredia CryoStar NX50	Epredia (Fisher UK)	Cat# 15320755
Nikon Ti2 Widefield/CSU-W1 Spinning Disk Confocal	Nikon	N/A

## Materials and equipment

**Table:** 1M Tris

Reagent	Final concentration	Amount
Tris base	1M	30.3 ml
MilliQ water	N/A	up to 250 ml after adjusting pH
Total	N/A	250 ml

Store at room temperature for up to 12 months.

***Note:*** Dissolve Tris base in 150 ml of MilliQ water and adjust the pH to 7.5 by adding approximately 18 ml of 37% hydrochloric acid. Top up the solution with MilliQ water to reach a final volume of 250 ml.

**Table:** 2M NaCl

Reagent	Final concentration	Amount
Sodium chloride	2M	11.69 g
MilliQ water	N/A	100 ml
Total	N/A	100 ml

Store at room temperature for up to 6 months.

**Table:** 10% SDS

Reagent	Final concentration	Amount
Sodium dodecyl sulfate (SDS)	10%	10 g
MilliQ water	N/A	100 ml
Total	N/A	100 ml

Store at room temperature for up to 12 months.

**CRITICAL:** SDS is harmful if swallowed and causes skin irritation and eye damage. In powder form, it is hazardous by inhalation. When handling SDS, wear lab coat and gloves and work in a chemical fume hood to prevent inhalation.

**Table:** DNA extraction buffer

Reagent	Final concentration	Amount
Tris base pH 7.5 1M	0.2M	20 ml
UltraPure™ EDTA 0.5M, pH 8.0	0.025M	5 ml
Sodium chloride 2M	0.25M	12.5 ml
MilliQ water	N/A	57.5 ml
Sodium dodecyl sulfate 10%	0.5%	5 ml
Total	N/A	100 ml

Store at room temperature for up to 6 months.

***Note:*** Add SDS once the other components are well mixed and dissolved.

**Table:** 50X TAE

Reagent	Final concentration	Amount
Tris base pH 7.5	2M	242 g
Acetic acid	1M	57.1 ml
EDTA 0.5M, pH 8.0	50 mM	100 ml
MilliQ water	N/A	842.9 ml
Total	N/A	1L

Store at room temperature for up to 12 months.

***Note:*** Autoclave the 50X stock solution before usage and use as 1X for gel electrophoresis.

## Step-by-step method details

### Axolotl skin swabbing

**Timing: ∼3–5 min per axolotl**

**Figure 1:**
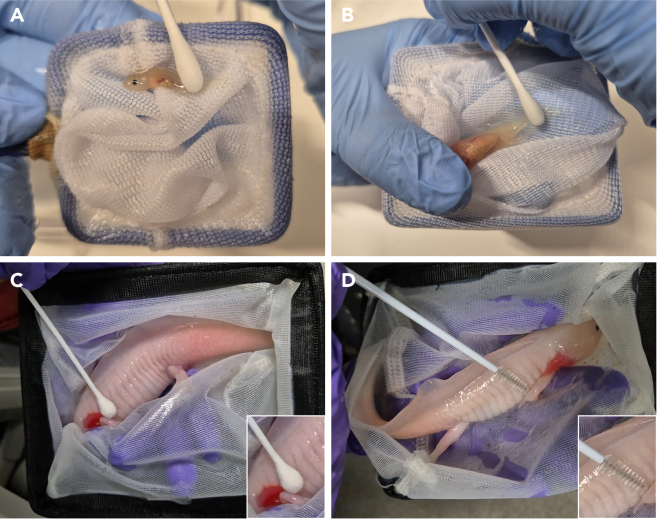
Skin swabbing of larval, juvenile and adult axolotls Skin swabbing of (A) 2.5 cm, (B) 4 cm, and (C) 25 cm axolotls (snout-to-tail tip) using cotton swabs. (D) Cytology brushes can also be used for skin swabbing.

Here, we describe how to collect mucus and skin cells from axolotls using cotton swabs (Figure 1). For a demonstration of the procedure on 2.5 and 25 cm axolotls refer to Methods Video S1 and Methods Video S2.

1. Preparation before swabbing
  a. Prepare DNA extraction buffer as described in Materials and Equipment.
  b. Prepare and label one DNA low-binding 1.5 ml microcentrifuge tube per axolotl.
  c. Prepare one sterile cotton swab per axolotl.
    **Alternatives**: We have also used cytology brushes with nylon bristles (Figure 1B) or more cost-effective autoclaved supermarket cotton swabs successfully. Because axolotls have very sensitive skin, we advise against using stiffer materials such as pipette tips for DNA sampling.
  d. If the axolotl is housed in a tank in a recirculating water system, close the water tap to prevent water spilling out of the tank while handling the axolotl.
  e. Remove all enrichment (hide-outs or plants) before fishing out the axolotl from the tank.
2. Skin swab axolotls
  ***Note:*** To be more efficient, we recommend that this step is performed by two people. Person 1 holds the axolotl in the net and performs the swabbing. Person 2 hands the sterile swab to Person 1 and places it in a tube once the swabbing is complete. This allows Person 1 to return the axolotl to its housing tank quickly and gently.
  a. Remove axolotl gently from housing tank using fish net.
  b. Hold the axolotl carefully and securely, ideally covering the head and eyes to keep it calm.
  c. Swab the skin by pressing a cotton swab gently but firmly against the axolotl trunk and tail skin as depicted in Figure 1 and Methods Video S1 and S2.
    i. Rotate the cotton swab while swabbing repeatedly.
    ii. Swab 5x each smaller (2.5-4 cm snout-to-tail tip) (Figures 1A and 1B) or large (≥4 cm) axolotl (Figures 1C and 1D).
      ***Note:*** The tails of small axolotls (<4 cm snout-to-tail tip) are quite fragile. To avoid damaging or excessively drying their tails, always pre-wet the cotton swab in clean axolotl water before swabbing. We recommend this for all axolotl sizes because adults are more robust but their skin remains sensitive to drying. If the tail or tail fin appears wrinkled the day after swabbing, refer to Troubleshooting Problem 1.
  d. Put the axolotl back into the housing tank.
    ***Note:*** Axolotls will swim actively when returned to the tank. Wait 10–15 s for the axolotl to settle before putting the enrichment back in the tank.
  e. Place cotton swabs into empty clean DNA low-binding 1.5 ml microcentrifuge tubes.
  f. Cut off the cotton swab stem to allow closing of the lid.
    ***Note:*** We recommend cutting the swab stem to allow the lid of the microcentrifuge tube to close fully while leaving enough length to pick the swab easily in later steps.
  g. Add enrichment back into the housing tank.

Methods Video S1. Skin swabbing a 2.5 cm axolotl using cotton swabs, related to Step 2

Methods Video S2. Skin swabbing a 25 cm axolotl using cotton swabs, related to Step 2

### DNA extraction and precipitation

**Timing: 2 h**

This step describes SDS- and salt-based DNA extraction from axolotl mucus and skin cells collected on cotton swabs and DNA precipitation using isopropanol.

3. DNA extraction.
  a. Cool isopropanol at −20^o^C (at least 10–15 min before DNA precipitation).
  b. Pre-warm DNA extraction buffer to 55°C (see materials and equipment) in a ThermoMixer.
    ***Note:*** Leave microcentrifuge tubes containing cotton swabs at room temperature until the DNA extraction buffer is pre-warmed. We have kept cotton swabs dry inside the tubes for a maximum of 1.5 h. Longer storage times may be compatible with DNA extraction but have not been tested.
  c. Add 400 μl of pre-warmed DNA extraction buffer to each tube.
  d. Vortex each tube for 10 s and incubate the samples at room temperature for 15 min.
  e. Remove swabs by thoroughly squeezing the swab against the side of the tube to recover as much solution as possible.
  f. Centrifuge samples in a benchtop centrifuge for 3 min at 12,000 × *g* to pellet debris.
4. DNA precipitation.
  a. Transfer the supernatant into a clean DNA low-binding 1.5 ml microcentrifuge tube and add pre-chilled isopropanol in a 1:1 ratio to precipitate DNA.
    ***Note:*** Samples may appear turbid.
  b. Invert microcentrifuge tubes 5x to mix the solutions and incubate samples at −80°C for 30 min or if needed, longer.
5. DNA collection and resuspension
  a. Let the samples defrost at room temperature for 2-3 min and then centrifuge them in a benchtop centrifuge for 10 min at 14,000 × *g*.
  b. Gently pour away the supernatant and keep the DNA pellet.
    ***Note:*** The DNA pellet most often appears as a large smear on the side of the tube. However, the lack of a visible pellet does not necessarily correlate with the lack of DNA.
  c. Add 190 μl of 70% ethanol along the side of the tube to wash the pellet and to remove residual isopropanol.
  d. Flick the samples gently and centrifuge in a benchtop centrifuge for 2 min at 14,000 × *g*.
  e. Remove the supernatant and dry the pellet fully in a ThermoMixer at 55°C for 5 min.
    **CRITICAL:** Remove the supernatant using a P200 pipette or smaller to remove as much solution as possible.
  f. Resuspend the dried pellet in 20 μl of nuclease-free water.
    ***Note:*** The pellet may not fully resuspend due to residual cotton fibers and aggregated cellular debris (Troubleshooting Problem 2). To mitigate this when using a swing-bucket centrifuge, rinse the sides of the tube with nuclease-free water to recover the entire pellet.
  g. Incubate the resuspended pellet in a ThermoMixer at 55°C for further 5 min.
  h. Before using the DNA for PCR amplification, vortex the samples for 3 s and spin down for 5 s in a minicentrifuge.
    ***Optional:*** Quantify DNA concentration using a spectrophotometer (Table 4).
    ***Note:*** If the DNA concentration is low (<20 ng/μl), refer to Troubleshooting Problem 3.
    **Pause point:** Store DNA at −20°C until use.

### PCR amplification of axolotl female-specific and non-sex specific DNA fragments

**Timing: 1 h**

This section outlines the steps for performing a polymerase chain reaction (PCR) to amplify female-specific and non-sex-specific genes of the axolotl genome. The PCR is set up using two primer pairs.^25^ The first primer pair (Axolotl-control-genotyping) amplifies the gene *Rcc2* present in both male and female axolotls, serving as an internal positive control. The second primer pair (Axolotl-female-genotyping) amplifies the female-specific gene *Atrw*. Together, these primer pairs allow male and female axolotls to be differentiated, with males yielding a single amplified fragment and females yielding two.

***Note:*** Perform the PCR according to the manufacturer’s protocol (Takara Bio USA Terra™ Direct PCR Polymerase Mix User Manual).

**CRITICAL:** Perform all the following steps on ice.

6. Prepare primers and PCR master mix
  a. Dilute primers (Table 1) to a final concentration of 10 μM using nuclease-free water.

**Table 1:** Primers for PCR

Primer name	Sequence	Tm (^o^C)
Axolotl-control-genotyping-fwd	GGTTTTAATTGTGATCAGTGGTACAG	63
Axolotl-control-genotyping-rev	AAAGAAATAAATAGGGCAAACAACAC	63
Axolotl-female-genotyping-fwd	AACCCAATACAAAAGGTAAACATGTAG	63
Axolotl-female-genotyping-rev	AGAAAGAGAAATTGGGCTTACTTTAAC	63
  b. Mix and flick the diluted primers gently and spin down for 5 s in a minicentrifuge before use.
  c. Prepare a PCR master mix (Table 2) and scale mixture according to the number of samples (i.e., axolotls swabbed) for each PCR reaction.
    ***Note:*** Do not add DNA template to the master mix. Mix and spin down the 2X Terra PCR Direct Buffer for 5 s in a minicentrifuge before use.
    ***Note:*** Terra Direct PCR Polymerase Mix is optimized for optimal, direct amplification from tissue samples, crude extracts, and dirty templates. The protocol may have to be optimized for other DNA polymerases.

**Table 2:** Reagents for the Terra Direct PCR master mix

Reagent	Amount	Final concentration	Volume (1 reaction)
Takara DNA Polymerase	–	–	1 μl
Axolotl-control-genotyping-fwd	15 pmol	0.3 μM	1.5 μl
Axolotl-control-genotyping-rev	15 pmol	0.3 μM	1.5 μl
Axolotl-female-genotyping-fwd	15 pmol	0.3 μM	1.5 μl
Axolotl-female-genotyping-rev	15 pmol	0.3 μM	1.5 μl
2X Terra PCR Direct Buffer (with Mg^2+^, dNTP)	–	–	25 μl
RNase-free ddH_2_O	–	–	16 μl
Total	–	–	48 μl
  d. Flick the PCR master mix gently and spin down for 5 s in a minicentrifuge.
  e. Add 2 μl of DNA template to each PCR tube.
    ***Note:*** The PCR works with a range of input DNA concentrations so, rather than normalizing concentrations across samples, we routinely use 2 μl of template directly. We have obtained robust amplification with DNA inputs ranging from ∼40 to ∼270 ng (see Table 4).
  f. Distribute 48 μl of PCR master mix to PCR tubes containing pre-aliquoted DNA template. This should make a total volume of 50 μl per reaction.
    ***Note:*** The manufacturer recommends a total PCR reaction volume of 50 μl, but we have obtained comparable results with a 25 μl reaction volume. If you are running a 25 μl reaction, scale all reagents to exactly half of the volumes listed in Table 2 and add 7 μl of RNase-free ddH2O per reaction. Keep the volume of DNA sample the same 2 μl.
  g. Flick PCR tubes gently to mix and spin down for 5 s in a minicentrifuge.
7. PCR setup.
  a. Run the PCR reaction according to the manufacturer’s protocol (Takara Bio USA Terra™ Direct PCR Polymerase Mix User Manual), see Table 3.

**Table 3:** PCR cycling conditions

Steps	Temperature	Time	Cycles
Initial Denaturation	98°C	2 min	1
Denaturation	98°C	10 s	35 cycles
Annealing	60°C	15 s	–
Extension	68°C	15 s	–
Hold and cooling	10°C	Infinite hold	1

**Pause point:** DNA can be stored at 4^o^C for 1-2 weeks before running the agarose gel.

### Agarose gel electrophoresis for visualization of PCR products

**Timing: 45 min**

This step includes the analysis of PCR-amplified products using agarose gel electrophoresis to sex genotype the axolotls. The first primer pair (Axolotl-control-genotyping) serves as an internal positive control that amplifies a 486 bp fragment of the *Rcc2* gene, present in both male and female axolotls. The second primer pair (Axolotl-female-genotyping) amplifies a 219 bp fragment of the female-specific gene *Atrw*. Female axolotls therefore yield two bands (486 bp and 219 bp) and male axolotls yield a single band (486 bp).

**Figure 2:**
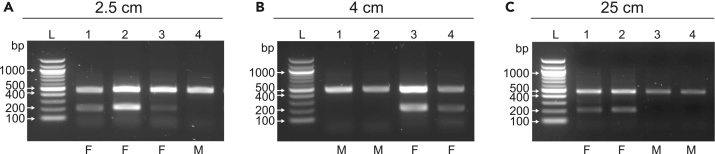
PCR-based sex genotyping of axolotls across life stages using skin swab-derived DNA PCR-amplified DNA products from (A) 2.5 cm, (B) 4 cm and (C) 25 cm axolotls (n = 4 per group). Upper bands (486 bp) represent the gene *Rcc2* present in both male and female axolotls, while lower bands (219 bp) represent the female-specific gene *Atrw*. A 100 bp DNA ladder (L) was loaded for size reference. M, male; F, female.

***Note:*** If no bands or only faint bands are visible on the agarose gel, refer to Troubleshooting Problem 4.

8. Agarose gel electrophoresis.
  a. Assemble the gel casting apparatus.
  b. Prepare a 1.5% agarose gel, by dissolving 1.5 g of agarose in 100 ml of 1X TAE (see materials and equipment). Heat the mixture for approximately 5 min in a microwave until the agarose is fully dissolved.
  c. Allow the agarose to cool down to approximately 37^o^C, then add 1:10 SYBR Safe DNA Gel Stain according to the manufacturer’s instructions (User Guide: SYBR Safe DNA Gel Stain).
  d. Pour the agarose into a gel casting tray with a fitted comb and allow the gel to polymerize at room temperature for approximately 30 min.
  e. In the meantime, mix each amplified DNA template with a gel loading dye (10X or 6X) to obtain a final volume of 10 μL per sample. If needed, mix the 100 bp DNA ladder with loading dye.
  f. Flick the tubes and spin down for 5 s in a minicentrifuge.
  g. Once the agarose gel has set, remove the comb, fill the electrophoresis tank with 1X TAE buffer and place the gel into the tank.
  h. Load the samples into the 1.5% agarose gel in 1X TAE and run the gel for 30 min at 100 V constant voltage.
  i. Visualize the bands on the gel on Optimal Auto-exposure (∼5 s) using ChemiDoc™ MP Imaging System (Bio-Rad) or equivalent system for imaging gels, Figure 2.

## Expected outcomes

This protocol uses skin swabbing to obtain DNA for genotyping and reliably assigns sex to 2.5 cm axolotls or larger (Figure 2), with minimal impact on their welfare.

DNA yields are sufficient across life stages and do not scale with animal size. In our hands, skin swabs yielded an average of 87.9 ng/μl of DNA from 2.5 cm axolotls, 118.9 ng/μl from 4 cm axolotls, and 39.3 ng/μl from 25 cm axolotls (Table 4). These yields are sufficient for PCR-based sex genotyping at every stage tested, but template input may need to be adjusted for other downstream applications (see limitations).

**Table 4:** DNA yields from axolotl skin swabs across life stages

Condition	Animal no.	Stroke number	Animal size (cm)	DNA concentration (ng/μl)	Average DNA concentration (ng/μl)	A260/280	A260/230
Skin swab	1	10	2.5	45	–	2.1	0.9
Skin swab	2	10	2.5	77.7	–	1.6	0.7
Skin swab	3	10	2.5	113.2	–	1.6	0.7
Skin swab	4	10	2.5	109.6	–	1.5	0.5
Skin swab	5	5	2.5	88.7	–	1.6	0.6
Skin swab	6	5	2.5	80.5	–	1.5	0.6
Skin swab	7	5	2.5	94.6	–	1.5	0.7
Skin swab 2.5 cm				45-113.2	87.0	1.5-2.1	0.5-0.9
Skin swab	1	10	4	69.1	–	1.8	0.8
Skin swab	2	10	4	135.9	–	1.4	0.7
Skin swab	3	10	4	78.8	–	1.8	0.7
Skin swab	4	10	4	81.1	–	1.9	0.8
Skin swab	5	10	4	230	–	1.7	0.7
Skin swab 4 cm				69.1-135.9	119.0	1.4-1.9	0.7-0.8
Skin swab	1	10	25	19.1	–	2.5	0
Skin swab	2	10	25	64.8	–	1.7	1
Skin swab	3	10	25	34.4	–	2.8	1
Skin swab	4	10	25	57.3	–	2.1	1
Skin swab	5	10	25	20.7	–	2.1	0
Skin swab 25 cm				20.7-64.8	39.3	1.7-2.8	0.3-0.8
Fin clip	1	–	7	52.6	–	1.7	1.1
Fin clip	2	–	7	196.1	–	1.5	1.1
Fin clip	3	–	7	147.6	–	1.5	0.8
Fin clip 7 cm				52-196.1	132.1	1.5-1.7	0.8-1.1

**Figure 3:**
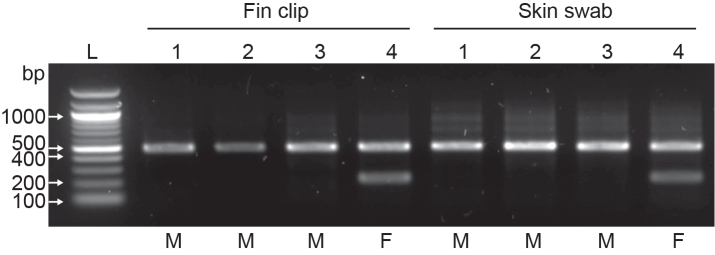
Skin swabbing and fin clipping generate equivalent sex genotyping results Axolotls (n = 4) were skin swabbed and then anaesthetized for fin clipping, with a 1 mm piece of tissue removed from the posterior tail fin. DNA was extracted from fin clip samples as in Keinath et al.^25^ DNA obtained from skin swabs and fin clips was PCR-amplified and visualized on an agarose gel. Upper bands (486 bp) correspond to the gene *Rcc2* present in both males and female axolotls, and the lower bands (219 bp) correspond to the female-specific gene *Atrw*. A 100 bp DNA ladder (L) was loaded for size reference. M, male; F, female.

Successful PCR amplification produces a clear banding pattern on a 1.5% agarose gel (Figures 2 and 3). Females yield two bands, at 486 bp (internal control, *Rcc2* fragment) and 219 bp (female-specific *Atrw* fragment), and males yield a single band at 486 bp (Figures 2 and 3). To benchmark this approach against the current standard, we processed paired skin swab and fin clip samples from the same axolotls and found that sex assignments matched in all cases tested (Figure 3). This demonstrates that skin swabbing is as a reliable, non-invasive alternative to fin clipping for routine sex genotyping in axolotl.

Skin swabbing also represents a welfare advantage. Axolotls tolerate the procedure well, resuming normal activity within 10-15 s of being returned to their housing tank. If needed, skin swabbing can be repeated on the same animal without observable adverse effects.

**Figure 4:**
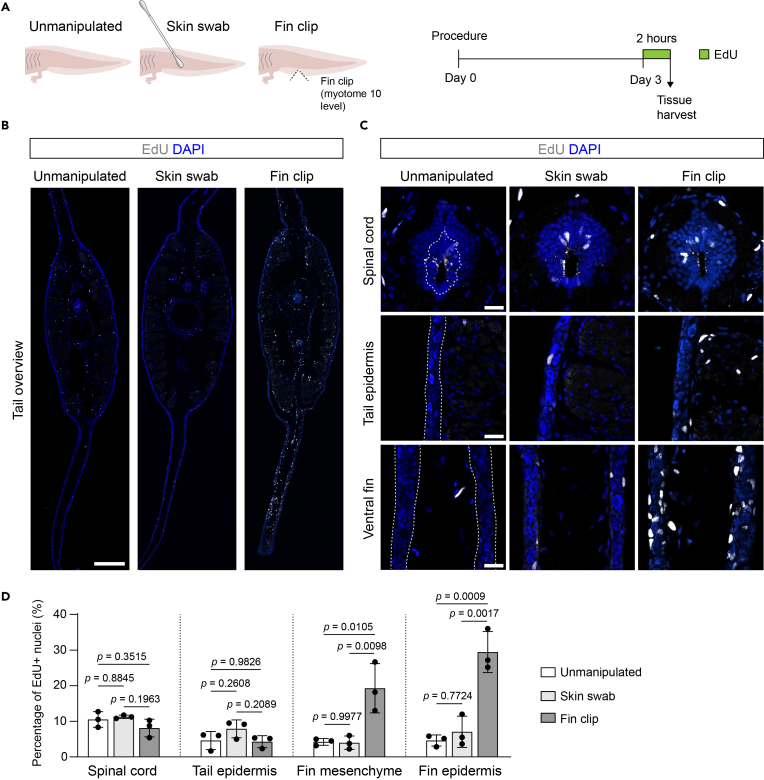
Fin clipping, but not skin swabbing, triggers an injury-induced proliferative response (A) Experimental design used to assess cell proliferation in response to different genotyping procedures. Axolotls were single-housed at 1.5 cm to ensure that all tissues remained intact and grown to 5 cm. Axolotls were fin clipped by removal of a 1 mm piece of tissue at the level of myotome 10 post-cloaca or skin-swabbed covering the trunk and tail (day 0). Unmanipulated axolotls were used as controls to set baseline proliferation. 3 days later, axolotls were fully anaesthetized in 0.01% benzocaine and injected intraperitoneally with 400 μM EdU (in PBS) at 20 μl/g body weight. EdU is a thymidine analogue that is incorporated into DNA during S phase of the cell cycle. FastGreen was added to the injection mix to aid visualization. Injected animals were kept out of water for a 2-minute recovery period on benzocaine-soaked towels and then returned to their tanks. Following a 2-h EdU pulse, tail tissue was harvested and processed for EdU detection and imaging. EdU detection was performed on 50 μm-thick cryosections with the Click-iT EdU Alexa Fluor 488 according to the manufacturer’s instructions (Click-iT EdU Imaging Kits). Tails from all three conditions were processed in parallel and sampled at the same anterior–posterior level (1.2 mm region beginning at myotome 8 post-cloaca). (B) Overview of cross-sections of tails of unmanipulated, skin swabbed and fin clipped axolotls. EdU labels cells duplicating their DNA during the 2-h EdU pulse. Images are single-plane, tiled images. EdU marks cells duplicating DNA; DAPI stains DNA. Scale bar, 1 mm. (C) Close-up views of the spinal cord, tail epidermis and ventral fin from the images in (B). Dashed outlines indicate the tissue compartments that were analyzed. Note that in ventral tail fin both the fin epidermis and the fin mesenchyme were analyzed. EdU marks cells duplicating DNA; DAPI stains DNA. Scale bar, 50 μm. (D) Quantification of the percentage of EdU-positive cells per compartment. Each dot represents one axolotl (n = 3 axolotls). Bars indicate the mean; error bars, SD; one-way ANOVA with Tukey’s multiple comparisons test (*p*, p-values; n.s. not significant).

The most critical advantage of skin swabbing is that it does not trigger an injury-like proliferative response. We compared cell proliferation in 5 cm axolotls that underwent skin swabbing, fin clipping, or no procedure (unmanipulated controls; Figure 4A). In the tail fin epidermis, cell proliferation was indistinguishable between skin-swabbed and unmanipulated axolotls but significantly higher in fin-clipped axolotls (7% and 5% compared with 30%; Figures 4B–4D). We observed a similar pattern in the tail fin mesenchyme (4% of the cells were proliferating in both skin-swabbed and unmanipulated axolotls compared with 19% in fin-clipped axolotls). Skin-swabbed axolotls showed a small, non-significant increase in tail epidermis proliferation relative to unmanipulated controls (Figures 4B–4D). This likely represents the need to replace the small number of epidermis cells lost with swabbing. In the deeper ependymal cell layer of the spinal cord, cell proliferation levels were indistinguishable across all three groups (Figures 4B–4D). Together, these data show that fin clipping induces a measurable proliferative response, while skin swabbing does not. These outcomes are likely to be applicable to axolotls of all sizes. Unlike fin clipping, which removes a piece of tissue, skin swabbing only collects superficial mucus and skin cells without tissue removal, making it an inherently less invasive procedure. We also do not observe adverse effects when skin swabbing axolotls of different sizes. These findings have direct and important implications for regeneration research. Because assigning sex to axolotls of most experimental sizes requires genotyping and the DNA sample is traditionally obtained by fin clipping, sexing the animals introduces a proliferative response that can confound cell proliferation readouts in regeneration studies. Skin swabbing circumvents this, enabling sex genotyping without interfering with regenerative processes. Importantly, sex differences are well documented in regeneration studies^35^^,^^36^^,^^37^ but remain largely unexplored in axolotls. This protocol makes it now feasible to study sex as a biological variable in axolotl research.

## Quantification and statistical analysis

Figure 4 images were analyzed in Fiji and cells were counted using the Cell Counter plugin.^34^ For quantification of EdU-positive cells across skin swabbed, fin clipped, and unmanipulated axolotls, tail cross-sections were divided into four compartments: ventral fin epidermis, ventral fin mesenchyme, tail epidermis, and spinal cord (Figures 4B and 4C). Within each compartment and cross-section, 200 cells were selected based on DAPI-stained nuclei and the percentage of these nuclei that were positive EdU quantified. 3-4 cross-sections from each axolotl were quantified or between 600-800 cells per compartment and axolotl. For spinal cord counts, the percentage of EdU-positive cells was quantified within the ependymal cell layer. Ependymal cells were identified based on their location lining the spinal cord central canal and elongated nuclear morphology. For fin and tail epidermis counts, both left and right sides were quantified. For fin mesenchymal cell counts, all cells in the space between the two epidermal layers were included in the analysis.

No statistical method was used beforehand to determine sample size. The investigators were not blinded, and no data points were excluded. Data is represented as mean ± SD of 3-4 sections per axolotl, with 3 axolotls per experimental group.

Statistical analysis and plots were performed in GraphPad Prism 11. Statistical significance was determined using a one-way ANOVA with Tukey’s multiple comparisons test (*p*, p-values).

## Limitations

Skin swabbing, while offering a less invasive alternative to fin clipping for axolotl genotyping, presents several limitations. Although skin swabs generally capture less genetic material than fin or toe clipping,^30^^,^^31^ our protocol yields DNA concentrations sufficient for routine genotyping and broadly comparable to fin clipping (Figure 3). As some yield variability is expected between samples, template input volumes should be empirically optimized when establishing the protocol in a new laboratory. Unlike fin clipping, which recovers a defined amount of tissue, swab-derived DNA is sensitive to swabbing pressure, stroke number, anatomical region sampled, and the speed of downstream processing. We recommend training all users to a standardized technique and processing swab and fin clip samples from the same axolotl in parallel during initial setup. It is also important to note that genotyping protocols optimized for fin clip-derived DNA may not transfer directly to swab-derived DNA, and PCR cycling conditions and template input volumes should be re-optimized accordingly.^26^^,^^27^^,^^38^

## Troubleshooting

### Problem 1

Skin lesions after swabbing (related to Step 2).

### Potential solution

Over-swabbing or excessive swabbing pressure can disrupt the mucosal barrier of the axolotl skin and cause the tail fin to wrinkle. To minimize potential damage, pre-wet swab and find the minimum number of strokes and pressure that yield enough DNA for the PCR reaction. We suggest following our recommendations as a starting point. Because the tail of larval axolotls (∼2.5 cm) is more delicate, swab only their head and trunk.

### Problem 2

The pellet cannot be fully resuspended because it contains residual cotton fibers (related to Step 5).

### Potential solution

Cotton swabs can shed some fibers in the DNA extraction buffer that co-pellet with cellular material during centrifugation, interfering with resuspension and downstream extraction. Centrifuge longer or at higher speed to obtain cleaner supernatant in Step 3. Alternatively, cytology brushes with nylon bristles (Figure 1B) do not shed fibers and may release cells more cleanly.

### Problem 3

Low DNA concentration (related to Step 5).

### Potential solution

The number of strokes or swabbing pressure may be insufficient. We recommend standardizing the number of strokes and swabbing pressure for each axolotl size. Ensure swabs are processed on the day of collection to minimize DNA degradation.

### Problem 4

The PCR bands in the agarose gel are too faint or there are no bands (related to Step 8).

### Potential solution

The amount of DNA/template for the PCR reaction is likely too low. First, confirm DNA concentration and purity in a NanoDrop or equivalent (>20 ng/μl). If the DNA concentration is too low, try swabbing more to collect more DNA, rotating the cotton swab along the whole trunk and tail. If A260/280 or A260/230 ratios are off, the PCR reaction could be overloaded because samples contain impurities that include PCR inhibitors. In this case, try diluting the amount of starting material to reduce the concentration of inhibitors in the PCR reaction.

## Resource availability

### Lead contact

Further information and requests for resources and reagents should be directed to and will be fulfilled by the lead contact, Aida Rodrigo Albors (aida.rodrigo-albors@ed.ac.uk).

### Technical contact

Technical questions on executing this protocol should be directed to and will be answered by the technical contact, Laura Isabella Arbanas (l.i.arbanas@ed.ac.uk).

### Materials availability

This study did not generate new unique materials or reagents.

### Data and code availability

This study did not generate datasets or code.

## Acknowledgments

We thank the BVS Aquatics Facilities team for outstanding axolotl care. We also thank Ruolan Zhou for help with axolotl care. We are thankful to all members of the Rodrigo Albors lab for feedback and to Yuka Taniguchi and Prayag Murawala for sharing with us a first protocol for axolotl sex genotyping. This work was supported by the 10.13039/100010269Wellcome Trust (Wellcome Career Development Award 304201/Z/23/Z to A.R.A.), a Chancellor’s Fellowship from The 10.13039/501100000848University of Edinburgh to A.R.A., the 10.13039/501100000268Biotechnology and Biological Sciences Research Council (BBSRC) (grant no. UKRI2241 to A.R.A.), and a BBSRC EastBio PhD studentship to L.I.A. This work was also supported by funding for the Wellcome Discovery Research Platform for Hidden Cell Biology (226791) and we gratefully acknowledge support from the Light Microscopy core.

## Author contributions

A.R.A. conceived the project and acquired funding. L.I.A. with support from A.R.A. performed the experiments. A.R.A. supervised the work. L.I.A. and A.R.A. wrote and revised the manuscript. All authors approved the manuscript.

## Declaration of interests

The authors declare no competing interests.
